# Supplementary material for: Evaluation of Bronopol and Disulfiram as Potential Candidatus Liberibacter asiaticus Inosine 5′-Monophosphate Dehydrogenase Inhibitors by Using Molecular Docking and Enzyme Kinetic
Source: Molecules. 2020 May 14;25(10):2313. doi: 10.3390/molecules25102313 (PMC7287799; doi:10.3390/molecules25102313)
Supplement: Supplementary file 1 [file molecules-25-02313-s001.pdf]

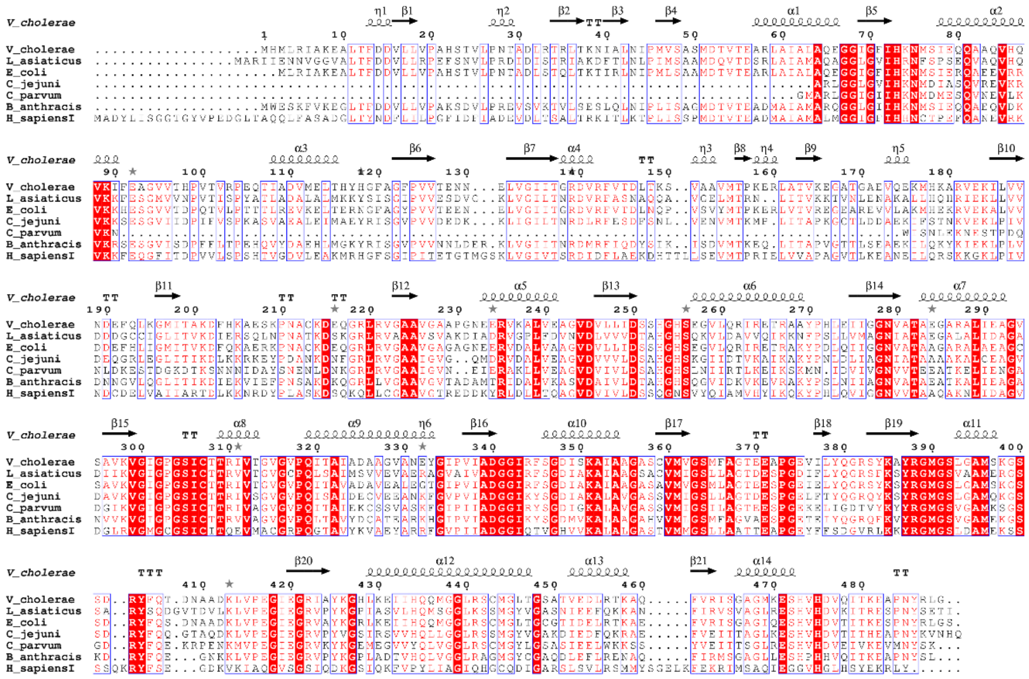

Figure S1. Multiple sequence alignment of IMPDHs. The sequences used in the alignment include *Vibrio cholera* O1 biovar; *Candidatus* Liberibacter asiaticus. strain psy62; *E. coli* str. K-12; *Campylobacter jejuni* subsp. Jejuni; *Cryptosporidium parvum*; *Bacillus anthracis* str. Ames; and *Homo sapiens* I.

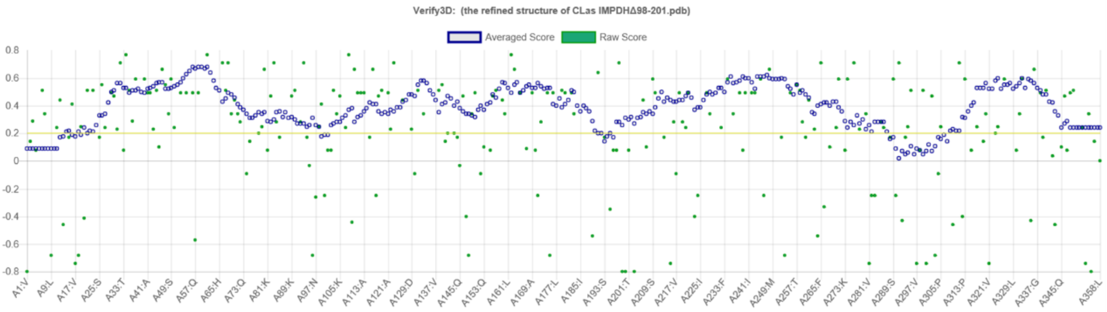

Figure S2. Verify 3D results of the refined *C*Las IMPDHΔ98-201.

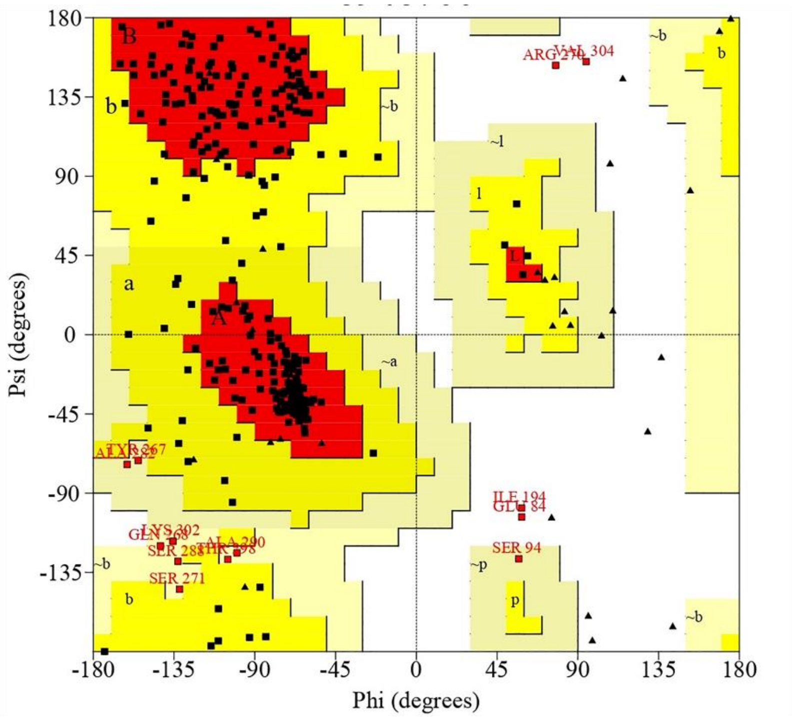

Figure S3. Ramachandran plot of *C*Las IMPDHΔ98-201 by PROCHECK.

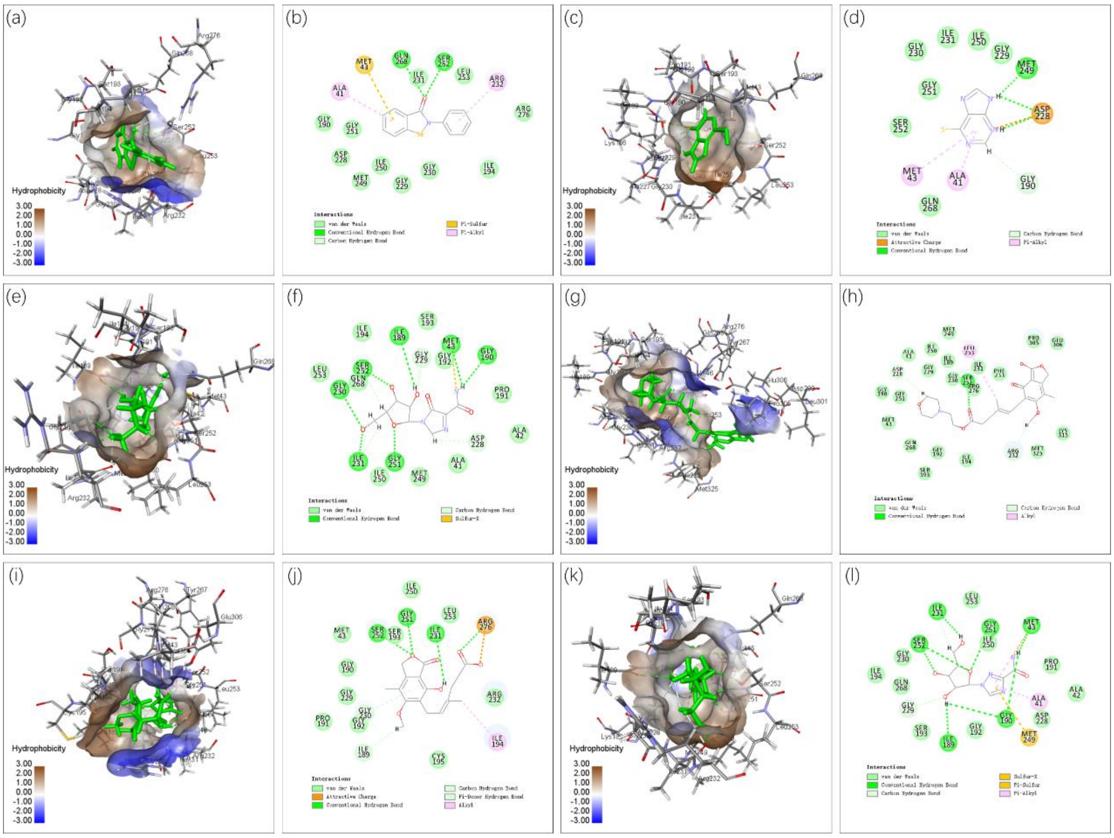

Figure S4. Molecular docking of *C*Las IMPDHΔ98-201 and the moleculars.

(a): 3-D details of *C*Las IMPDHΔ98-201 and ebselen (green) interaction; **(b):** 2-D interaction

details of *C*Las IMPDHΔ98-201 and ebselen; **(c):** 3-D details of *C*Las IMPDHΔ98-201 and Mercaptopurine (green) interaction; **(d):** 2-D interaction details of *C*Las IMPDHΔ98-201 and Mercaptopurine; **(e):** 3-D details of *C*Las IMPDHΔ98-201 and Mizoribine (green) interaction; **(f):** 2-D interaction details of *C*Las IMPDHΔ98-201 and Mizoribine; **(g):** 3-D details of *C*Las IMPDHΔ98-201 and Mycophenolate_mofetil (green) interaction; **(h):** 2-D interaction details of *C*Las IMPDHΔ98-201 and Mycophenolate_mofetil; **(i):** 3-D details of *C*Las IMPDHΔ98-201 and Mycophenolic_acid (green) interaction; **(j):** 2-D interaction details of *C*Las IMPDHΔ98-201 and Mycophenolic_acid; **(k):** 3-D details of *C*Las IMPDHΔ98-201 and Ribavirin (green) interaction; **(l):** 2-D interaction details of *C*Las IMPDHΔ98-201 and Ribavirin.

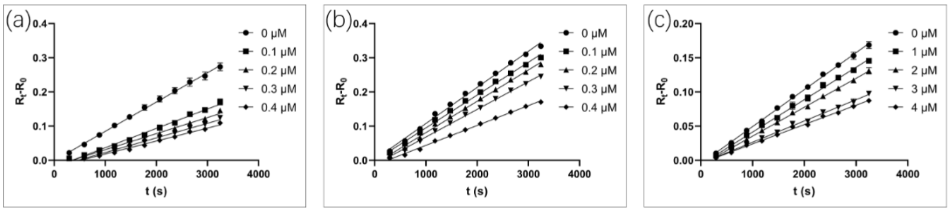

Figure S5. Bronopol, disulfiram and ebselen reversibly inhibit *C*Las IMPDHΔ98-201. Elongating the reaction time, no exponential enzyme decay observed. Substrate concentrations are 500 μM IMP and 1 mM NAD^+ for *C*Las IMPDHΔ98-201. **(a):** bronopol; **(b):** disulfiram; **(c):** ebselen.

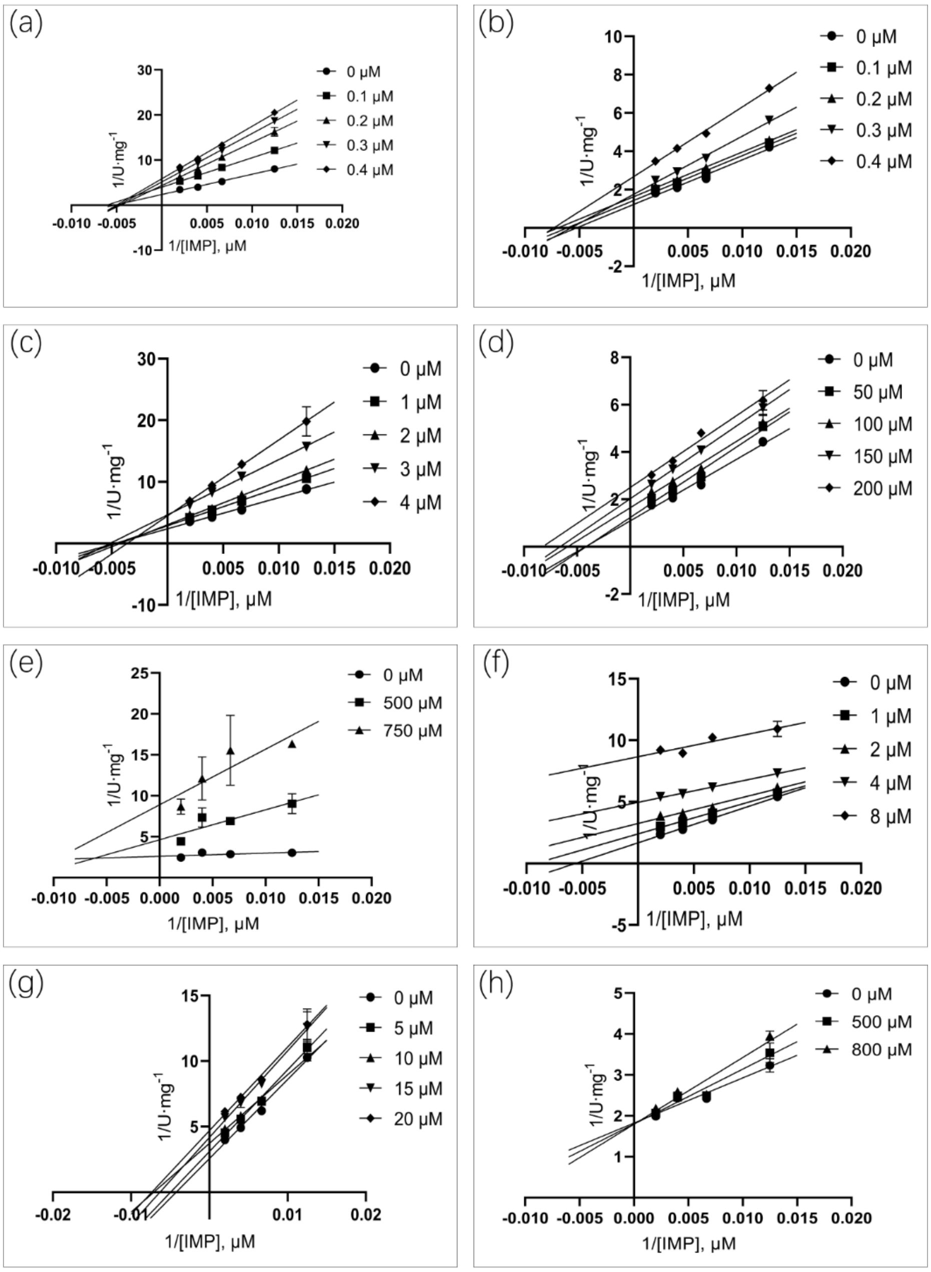

Figure S6. Mechanism of *C*Las IMPDHΔ98-201 inhibition by small molecules was studied against varying concentrations of IMP. The data is plotted using the Lineweaver-Burk equation. **(a):** bronopol; **(b):** disulfiram; **(c):** ebselen; **(d):** mercaptopurine; **(e):** mizoribine; **(f):** mycophenolic acid; **(g):** mycophenolate mofetil; **(h):** ribavirin.

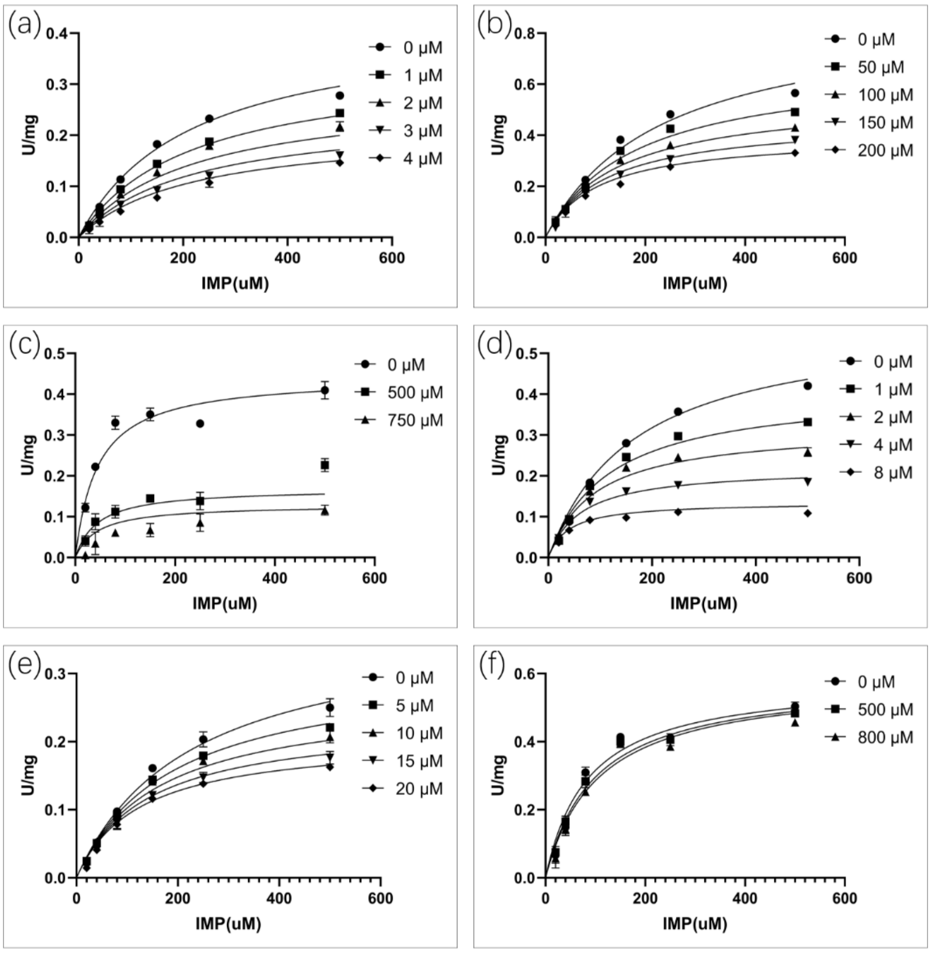

Figure S7. Inhibition kinetics at different concentrations of compounds by varying the IMP concentrations at a fixed NAD⁺ concentration. (a): ebselen; **(b):** mercaptopurine; **(c):** mizoribine; **(d):** mycophenolic acid; **(e):** mycophenolate mofetil; **(f):** ribavirin.

Table S1. Parameters for molecular docking experiments performed with CDOCKER.

input site sphere	Parameter value	input site sphere	parameter value
x	14.56	keep hydrogrns	FALSE
y	48.79	max conformation hits	30
z	15.78	max start conformations	1000
number of hotpot	100	steric fraction	0.1
docking tolerance	0.25	final cluster radius	0.5
Docking preference	user specified	apolar SASA cutoff	15
max hits to save	10	polar SASA cutoff	5
max number of hits	100	surface grid steps	18
minimum CDOCKER score	100	conformation method	Best
final score cutoff	0.5	minimization algorithm	do not minimize

max BFGS steps	50	parallel processing	FALSE
rigid optimization	FALSE		

Table S2. Kinetic parameters of bacterial IMPDHΔ constructs.

**Enzyme**	**K_{cat} S^{-1}**	**IMP,K_m μM**	**NAD^+, K_m μM**	**NAD^+, K_{ii} mM**
*C*Las IMPDHΔ98-201	7.2±0.2	181±19	318±24	7.3±1.1
*Ba*IMPDHΔ95-200[a]	6.1±0.3	61±4	560±50	5.3±0.7
*Ba*IMPDHΔ92-220[a]	4.5±0.2	150±20	460±50	3.8±0.5
*Cj*IMPDHΔ92-195[a]	1.9±0.1	27±3	520±70	6.4±0.7
*Cl*pIMPDHΔ89-215[a]	1.8±0.1	49±4	510±60	9±1
*Vc*IMPDHΔ91-219[a]	5.2±0.3	87±13	1100±100	13±3

[a] Data are from Makowska-Grzyska et al. (2015).
